# Success rates with nicotine personal vaporizers: a prospective 6-month pilot study of smokers not intending to quit

**DOI:** 10.1186/1471-2458-14-1159

**Published:** 2014-11-08

**Authors:** Riccardo Polosa, Pasquale Caponnetto, Marilena Maglia, Jaymin B Morjaria, Cristina Russo

**Affiliations:** Centro per la Prevenzione e Cura del Tabagismo (CPCT), Azienda Ospedaliero-Universitaria “Policlinico-Vittorio Emanuele”, Università di Catania, Catania, Italy; Dipartimento di Biomedicina Clinica e Molecolare, Università di Catania, Azienda Ospedaliero-Universitaria “Policlinico-Vittorio Emanuele”, Università di Catania, Catania, Italy; Department of Academic Respiratory Medicine, Hull York Medical School, University of Hull, Castle Hill Hospital, Castle Road, Cottingham Kingston, HU16 5JQ UK; UOC di Medicina Interna e d’Urgenza, Edificio 4, Piano 3, AOU “Policlinico-V. Emanuele”, Via S. Sofia 78, 95123 Catania, Italy

**Keywords:** Smoking cessation, Smoking reduction, Electronic cigarette, Personal vaporizers, Efficacy, Safety, Tobacco harm reduction

## Abstract

**Background:**

Electronic cigarettes (e-Cigs) are an attractive long-term alternative nicotine source to conventional cigarettes. Although they may assist smokers to remain abstinent during their quit attempt, studies using first generation e-Cigs report low success rates. Second generation devices (personal vaporisers - PVs) may result in much higher quit rates, but their efficacy and safety in smoking cessation and/or reduction in clinical trials is unreported.

**Method:**

We conducted a prospective proof-of-concept study monitoring modifications in smoking behaviour of 50 smokers (unwilling to quit) switched onto PVs. Participants attended five study visits: baseline, week-4, week-8, week-12 and week-24. Number of cigarettes/day (cigs/day) and exhaled carbon monoxide (eCO) levels were noted at each visit. Smoking reduction/abstinence rates, product usage, adverse events and subjective opinions of these products were also reviewed.

**Results:**

Sustained 50% and 80% reduction in cigs/day at week-24 was reported in 15/50 (30%) and 7/50 (14%) participants with a reduction from 25cigs/day to 6cigs/day (p < 0.001) and 3cigs/day (p < 0.001), respectively. Smoking abstinence (self-reported abstinence from cigarette smoking verified by an eCO ≤10 ppm) at week-24 was observed in 18/50 (36%) participants, with 15/18 (83.3%) still using their PVs at the end of the study. Combined 50% reduction and smoking abstinence was shown in 33/50 (66%) participants. Throat/mouth irritation (35.6%), dry throat/mouth (28.9%), headache (26.7%) and dry cough (22.2%) were frequently reported early in the study, but waned substantially by week-24. Participants’ perception and acceptance of the products was very good.

**Conclusion:**

The use of second generation PVs substantially decreased cigarette consumption without causing significant adverse effects in smokers not intending to quit.

**Trial registration:**

(ClinicalTrials.gov Identifier: NCT02124200)

## Background

Most smokers want to quit and make attempts to do so, but the majority of these attempts fail largely because the powerful addictive qualities of nicotine and non-nicotine sensory and behavioural cues [1, 2]. For those willing to quit, combination of pharmacotherapy and intensive behavioural intervention for smoking cessation can support their quit attempts and can double or triple quit rates [3, 4]. However, outside the context of a rigorous clinical trial (where there tends to be intensive support), their efficacy rates are somewhat lower, not exceeding 10% [5, 6]. Several population-based studies evaluating the value of pharmacotherapy outside the context of clinical trials have also shown modest quit rates with antismoking medications [7–9]. Consequently, the need for novel and more efficient approaches to smoking cessation interventions is unquestionable.

**Figure 1:**
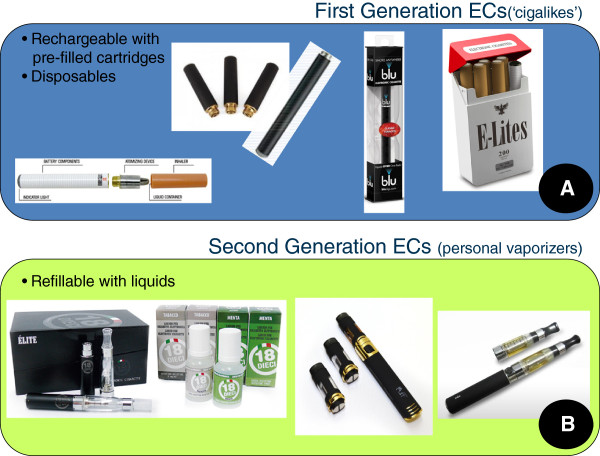
**E-cigarettes are battery-powered electronic nicotine delivery device (ENDD) resembling a cigarette designed for the purpose of providing inhaled doses of nicotine by way of a vaporized solution to the respiratory system.** These devices provide a flavor and physical sensation similar to that of inhaled tobacco smoke, while no smoke or combustion is actually involved in its operation. For the purpose of the current study, E-cigarettes can be distinct in first generation **(A)** and second generation devices **(B)**. First-generation devices, generally mimic the size and look of conventional cigarettes and consist of small lithium batteries and cartomizers (i.e. cartridges, which are usually prefilled with a liquid that bathes the atomizer); their batteries may be disposable (to be used once only) or rechargeable. Second-generation devices, consist mainly of higher-capacity (larger) rechargeable lithium batteries and atomizers with the ability to refill them with liquid (sold in separate vials). In the most recent atomizers you can simply change the atomizer head (resistance and wick) while keeping the body of the atomizer, thus reducing the operating costs. They do not resemble conventional cigarettes.

Electronic cigarettes (e-Cigs) are an attractive long-term alternative source of nicotine to conventional cigarettes because of their many similarities with smoking [10, 11]. Moreover, users report buying them to reduce cigarette consumption, to relieve tobacco withdrawal symptoms, to quit, and to continue having a ‘smoking’ experience, but with reduced health risks [12, 13]. Two RCTs have recently reported disappointingly low quit rates with e-Cigs; 4–8.7% for the ECLAT study in Italy [14] and 4–7.3% for the ASCEND study in New Zealand [15]. A likely explanation for the poor results is to be attributed to the unsatisfactory quality of the products under investigation, essentially first-generation cig-alike devices consisting of small rechargeable batteries and disposable cartridges (Figure 1A). Their lithium battery allowed only a limited number of puffs and required frequent recharging. Reliability was questionable due to the high frequency of technical malfunctions. Moreover, both products were not very efficient at delivering nicotine [16]. Presumably, these products were not performing adequately as cigarette substitutes.

Second-generation devices (or personal vaporizers (PVs)) are equipped with higher-capacity lithium batteries, much efficient vaporizing systems and cartridges that can be refilled with liquid solutions mainly consisting of propylene glycol (PG), glycerol, distilled water, flavourings and nicotine (i.e. e-Liquid) (Figure 1B). These devices assent to a more fulfilling vaping experience with the choice of an extensive number of puffs and e-liquid aromas, and thicker vapor [12, 13]. Moreover, nicotine delivery to the bloodstream using second-generation devices is consistently superior compared to “cig-alikes” [17, 18].

Consequently, PV use may result in higher quit rates compared to “cig-alikes”. With this in mind, we designed a prospective proof-of-concept study to monitor possible modifications in daily cigarette consumption in smokers switching to second generation PVs focusing on smoking reduction and abstinence. We also monitored product use and adverse events and evaluated participants’ perception and acceptance of the product.

## Methods

### Participants

Healthy smokers 18–60 years old, smoking ≥15 conventional cigarettes per day (cig/day) for at least 10 years were recruited using anti-smoking leaflets and by an approved kiosk located in the atrium of the university hospital (AOU ‘Policlinico-V.Emanuele’) promoting smoking cessation services at CPCT (Centro per la Prevenzione e Cura del Tabagismo, Università di Catania, Italy).

**Figure 2:**
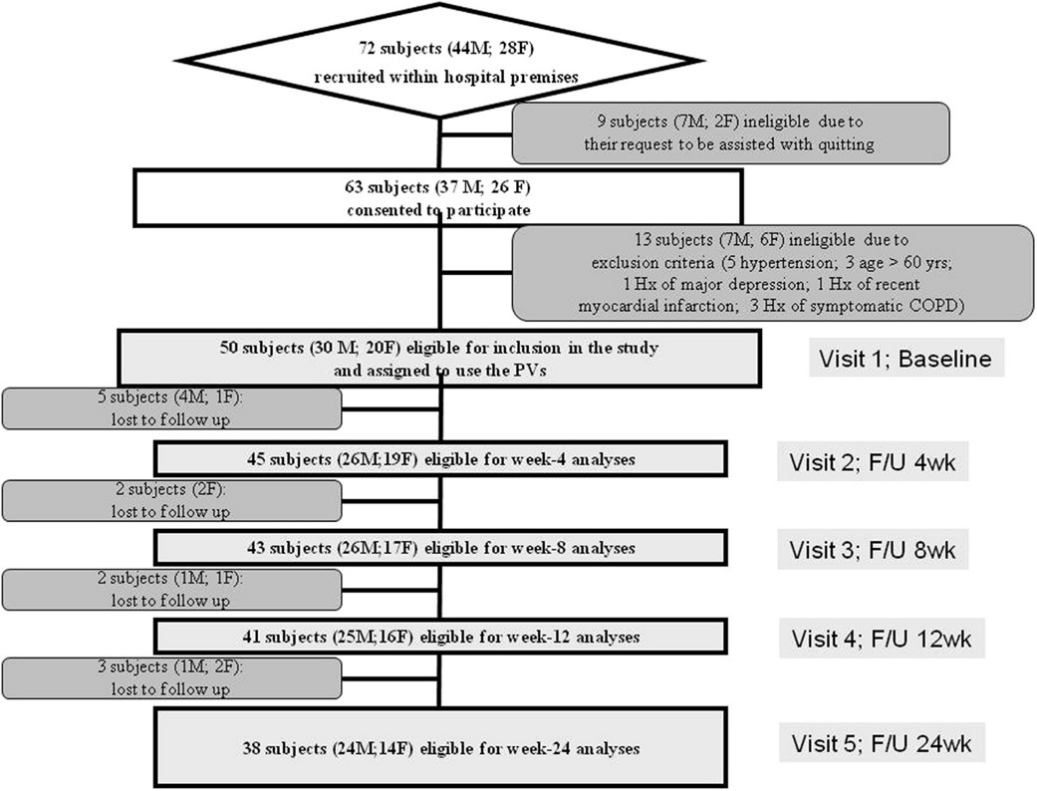
**Recruitment and flow of subjects within the study.** A total of 72 subjects with specifically predefined smoking criteria (smoking ≥15 cig/day for at least 10 years) responded to the advert; of these, 9 subjects were not included in the study because they spontaneously seek assistance with quitting (these were then invited to attend the local smoking cessation clinic, which offers standard support with cessation counselling and pharmacotherapy for nicotine dependence). The remaining 63 subjects consented to participate into the study; of these, 13 were not considered eligible because of the exclusion criteria. In the end, 50 volunteers were included in the study and were issued with a second generation PV kit with a full supply of tobacco aroma e-liquid containing 9 mg/ml nicotine. By the end of the study, a total of 12 subjects were lost to follow-up due to failure of attending their control visits. Overall 38 participants were available for analyses at week-24 follow-up visit.

Intent to quit smoking or wishing to do so in the next 30 days was investigated at screening using 2 questions: “*Do you intend to quit in the next 30 days?*” and “*Are you interested in taking part in one of our smoking cessation programs?*”. If subjects answered “*no*” to both questions, then they were considered eligible for inclusion. If they answered “*yes*”, they were invited to attend our standard smoking cessation program; of all the subjects approached, 9 (7 M, 2 F) requested to attend professional smoking cessation services and were excluded from the study (Figure 2).

None of the participants reported a history of alcohol and illicit drug use, major depression or other psychiatric conditions. The study protocol was approved by the University of Catania Ethics Review Board and subjects gave written consent prior to participation.

### Study design and baseline measures

Eligible participants were invited to use a second generation device (EGO/CE4 model, filled with tobacco aroma e-Liquid containing 9 mg/ml nicotine) and were followed-up prospectively for 6 months. They attended a total of five study visits at our smoking cessation clinic (CPCT, Università di Catania, Italy) comprising of a baseline visit and four follow-up visits at week-4, 8, 12, and 24 (Figure 2).

At baseline, basic demographic and smoking history were taken together with scoring of their level of nicotine dependence by means of Fagerstrom Test of Nicotine Dependence (FTND) questionnaire [19]. Subjective ratings of depression were assessed with the Beck Depression Inventory (BDI) [20]. Additionally, levels of carbon monoxide in exhaled breath (eCO) were measured using a portable device (Micro CO, Micro Medical Ltd, UK).

Participants were then given a second generation PV and a full supply of tobacco aroma e-Liquid containing 9 mg/ml nicotine for 4 weeks (14 vials in total). Commercially available PV kits (EGO/CE4 model with a rechargeable 3.7 V - 650mAh lithium-ion battery, charger, and CE4 atomizer) and e-Liquids (Tuscan Reserve; FlavourArt – Italy, http://www.flavourart.it, and Calliope; DEA Flavour – Italy, http://www.flavourart.it; both consisting of a similar PG/VG base) were purchased from local vapeshops out of a generous grant by LIAF (Lega Italiana Anti Fumo). These products are among the most popular in Italy and were selected because of positive reviews in specialized forums. Participants were instructed how to charge, fill, activate and use the e-Cig. Key troubleshooting was addressed and phone numbers were supplied for both technical and medical assistance.

Participants were permitted to use the study products *ad libitum* (up to a maximum of 5 ml/day; i.e. half vial) in the anticipation of reducing the number of cig/day smoked, and to fill a 4-weeks’ study diary recording product use, number of conventional cigarettes smoked, and adverse events. The participants were invited to attend at week-4, week-8, and week-12 to have their eCO levels measured, and to return their study diaries and unused study products. At these visits participants received further free e-Liquid refills together with the study diaries for the residual study periods.

Participants returned for a final visit at week-24 during which product use (total e-Liquid volume per day and frequency of use), number of cig/day (from which smoking reduction and abstinence could be computed), eCO level and a subjective rating of the usefulness of the study products were assessed. For the latter, participants were asked to rate their level of satisfaction with the products compared to conventional cigarettes using a visual analogue scale (VAS) from 0 to 10 points (0 = being ‘completely unsatisfied’, 10 = ’fully satisfied’); on the same scale, they also rated helpfulness (in keeping them from smoking) and whether they would recommend the PV to a friend who wanted to stop/reduce smoking. Adverse events were obtained from their study diaries.

No emphasis on encouragement, motivation and reward for the smoking cessation-related efforts were provided during the study. Although participants were encouraged to use these products, they were told that they were at liberty to smoke their own brand conventional cigarettes as they wished.

### Study outcome measures

Sustained 50% reduction in the number of cig/day at week-24 from baseline (***reducers***) [21] was defined as sustained self-reported 50% reduction in the number of cig/day compared to baseline for the 30 days period prior to week-24 study visit (eCO levels were measured to verify smoking status and confirm a reduction compared to baseline).

Sustained 80% reduction in the number of cig/day (***heavy reducers***) and sustained smoking abstinence at week-24 from baseline (***quitters***) were defined as sustained self-reported 80% reduction in the number of cig/day compared to baseline and complete self-reported abstinence from tobacco smoking (not even a puff) for the 30 days period prior to week-24 study visit respectively. eCO levels were measured to verify smoking status and confirm a reduction compared to baseline for the former and an eCO concentration of ≤10 ppm for the quitters, respectively.

Smokers who failed to meet the above criteria at the final week-24 follow-up visit were categorized as reduction/cessation failures (***failures***).

Adverse events were obtained from study diaries; withdrawal symptoms were reviewed at each visit by asking about the presence/absence of irritability, restlessness, difficulty concentrating, increased appetite/weight gain, depression or insomnia.

### Statistical analyses

As this was a proof-of-concept pilot study no previous data for PVs could be used for power calculation. However, by considering the results from our previous smoking reduction and cessation study with first generation e-cigs (i.e. cig-alikes) [22], we estimated that a sample of 50 subjects would have been adequate.

Primary and secondary outcome measures were computed by including all enrolled participants - assuming that all those individuals who were lost to follow-up are classified as failures (intention-to-treat analysis). Parametric and non-parametric data were expressed as mean (±SD) and median (interquartile range (IQR)) respectively. Paired and unpaired non-parametric, and parametric data were analysed using Wilcoxon Signed rank test and Mann Whitney U test, and student’s t test respectively. Correlations were calculated using Spearman’s Rho correlation. Statistical methods were 2-tailed, and p values of <0.05 were considered significant.

## Results

### Participant characteristics

After considering study inclusion and exclusion criteria, a total of 50 (M 30; F 20; mean (±SD) age of 41 (±8.9) years) regular smokers (mean (±SD) pack/yrs of 31.3 (±13.9)) consented to participate and were included in the study (Table 1; Figure 2). Retention rate in this study was high, with thirty-eight (76%) participants completing all study visits and attending their final follow-up visit at week-24. Baseline characteristics of those who were lost to follow-up were not significantly different from participants who completed the study.

**Table 1:** **Baseline subjects demographics**

	Parameter	Mean (±SD)
Subjects eligible for inclusion (n = 50)		
	Age	41.0 (±8.9)
	Sex	30 M; 20 F
	Smoking Pack Years	31.3 (±13.9)
	FTND	6.0 (4, 7)*
	Beck Depression Inventory	6.5 (3, 13.5)*
	Cigarettes/day	25 (20, 30)*
	eCO	23 (17, 32.8)*
†Subjects available for week-24 analyses (n = 38)		
	Age	40.7 (±8.6)
	Sex	24 M; 14 F
	Smoking Pack Years	31.1 (±15)
	FTND	5.5 (3.3, 7)*
	Beck Depression Inventory	4 (1, 10.8)*
	Cigarettes/day	25 (20, 28.8)*
	eCO	22.5 (16.3, 32)*

*Abbreviations:* *SD* Standard Deviation, *M* Male, *F* Female, *FTND* Fagerstrom Test of Nicotine Dependence, *eCO* exhaled carbon monoxide, *IQR* interquartile range.

*Non-parametric data expressed as median (IQR).

†Subjects excluding those lost-to-follow-up.

#### Changes in smoking behaviour

**Figure 3:**
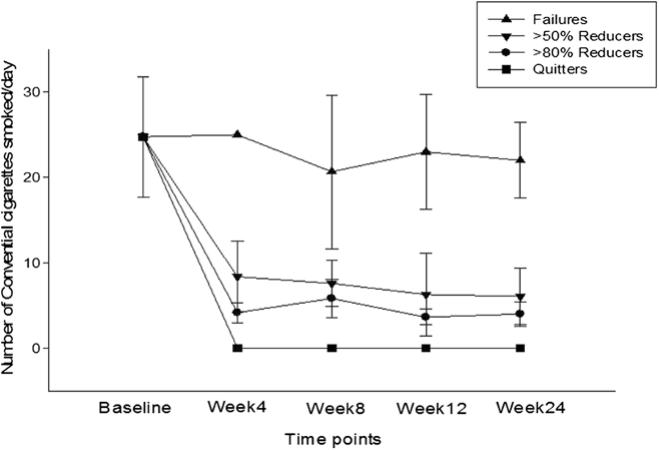
**Changes in the mean (±SD) number of conventional cigarettes use per day for each study subgroups throughout the study.**

**Figure 4:**
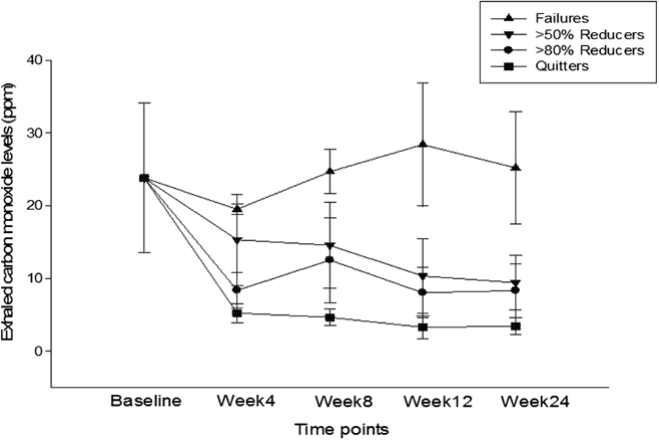
**Changes in the mean (±SD) exhaled carbon monoxide (ppm) for each study subgroups throughout the study.**

Participants’ smoking status at baseline and at 24-week is shown on Table 2. Taking the whole cohort of participants (n = 50), an overall 80% reduction in median cig/day use from 25 to 5 was observed by the end of the study (p < 0.001). Sustained 50% reduction in the number of cig/day at week-24 was shown in 15/50 (30%) participants, with a median of 25 cig/day (IQR 20, 30) decreasing significantly to 6 cig/day (IQR 3.5, 6) (p < 0.001). Of these tobacco smoke reducers, seven (14%) could be classified as sustained heavy reducers (at least 80% reduction in the number of cig/day) at week-24. They had a median consumption of 25 cig/day (IQR 18, 32.5) at baseline, decreasing significantly to 3 cig/day (IQR 3, 5) (p < 0.001). There were 18/50 (36%) quitters in total, with 15/18 (83.3%) still using their PVs by the end of the study. Overall, combined sustained 50% reduction and smoking abstinence was shown in 33/50 (66%) participants, with a median of 25 cig/day (IQR 20, 30) decreasing significantly to 3 cig/day (IQR 0, 5) (p < 0.001), which is equivalent to an overall 88% reduction. Details of mean conventional cigarette use and eCO levels throughout the study are shown in Figures 3 and 4, respectively.

**Table 2:** **Subjects characteristics at baseline and after 24 weeks of personal vaporiser use**

Parameter	At Baseline	At 24-Weeks Post PV use	***p value‡***
Sustained >50% (excluding quitters; includes sustained >80% reducers ) reduction in cigarette smoking (n = 15)			
Age	39.9 (±8.7)†		
Sex	10 M; 5 F		
Smoking Pack Years	31.1 (±16.5)†		
Cigarettes/day	25 (20, 30)*	6 (3.5, 6)*	<0.001
eCO	18 (13, 32.5)*	10 (7, 11.5)*	<0.001
Sustained >80% (excluding quitters) reduction in cigarette smoking (n = 7)			
Age	40.3 (±11.2)†		
Sex	6 M; 1 F		
Smoking Pack Years	32.6 (±20.6)†		
Cigarettes/day	25 (18, 32.5)*	3 (3, 5)*	<0.001
eCO	17 (13, 33)*	10 (5, 10)*	0.016
Sustained 100% (quitters) reduction in cigarette smoking (n = 18)			
Age	40.2 (±8.9)†		
Sex	12 M; 6 F		
Smoking Pack Years	30.9 (±13.5)†		
igarettes/day	25 (20.5, 25)*	0 (0, 0)*	<0.001
eCO	23 (17.5, 29.3)*	3 (2.3, 4)*	<0.001
Smoking Failure (<50% smoking reduction) (n = 5)			
Age	45.0 (±7.3)†		
Sex	2 M; 3 F		
Smoking Pack Years	32 (±18.5)†		
Cigarettes/day	20 (20, 25)*	20 (20, 20)*	0.732
eCO	18 (16, 32)*	28 (17, 31)*	0.819
Lost to Follow-up (n = 12)			
Age	42.2 (±10.2)		
Sex	6 M; 6 F		
Smoking Pack Years	31.9 (±10.6)		
Cigarettes/day	25 (20, 30)	N/A	N/A
eCO	24 (19.8, 34.5)	N/A	N/A

*Abbreviations*: *SD* Standard Deviation, *M* Male, *F* Female, *eCO* exhaled carbon monoxide.

‡p value – within group Wilcoxon Signed Rank Test.

†Parametric data expressed as mean (±SD).

*Non-parametric data expressed as median (interquartile range (IQR)).

#### Product use

**Figure 5:**
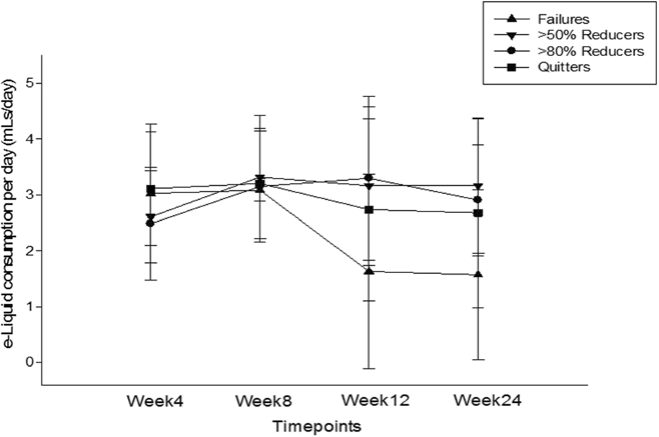
**Changes in the mean (±SD) daily e-Liquid consumption per day (mLs/day) for each study subgroups throughout the study.**

Details of median amount of e-Liquid (millitres/day (mLs/day)) used are shown on Figure 5. The reported use of e-Liquid used was very variable within and among failures, reducers, and quitters. For the whole group that completed the all the visits (n = 38) the median (IQR) usage over the 24 weeks was 2.85mLs/day (2.2, 3.9). Of note, the overall amount of e-Liquid consumed was marginally higher when these summary statistics were computed with the exclusion of the five participants who failed (failures) to give up smoking conventional cigarettes, i.e. reducers and quitters combined; the amount increasing to a median (IQR) of 3.2mLs/day (2.5, 3.9). Comparisons between overall e-Liquid use and main study outcomes (i.e. failures, reducers and quitters) are summarised on Table 3. In particular, no correlations were observed between daily consumption of e-Liquid and success rates; however, failures were consuming significantly less e-Liquid than reducers/quitters. Furthermore, correlations between e-Liquid use at week-4 and baseline FTND, pack/yrs and cig/day were weak and insignificant. Likewise, no significant relationships were observed between baseline FTND, pack/yrs and cig/day and e-Liquid use at all subsequent study visits.

**Table 3:** **Comparisons between average daily e-Liquid consumption (mLs/day) and study outcome measures**

	Failures (n = 15)	>50% Reducers (n = 7)	>80% Reducers (n = 7)	Quitters (n = 18)	>50% Reducers & Quitters (n = 33)
Median e-Liquid (mLs/day) use (IQR)	1.98 (1.4, 3.2)	3.03 (2.2, 3.9)	2.85 (2.2, 3.9)	2.85 (2.2, 3.2)	3.2 (2.5, 3.9)
‡p value VS Failures	-	<0.001	0.010	0.013	0.002

*Abbreviations:* *mL* millilitres, *n* number of subjects, *IQR* interquartile range.

‡p value – between group Mann Whitney U Test.

#### Adverse events

Frequently reported adverse events in regular PV users were throat/mouth irritation (35.6%), dry throat/mouth (28.9%), headache (26.7%) and dry cough (22.2%) (Table 4). These events were most commonly reported at the beginning of the study and appeared to wane spontaneously by the end of the study. Of note, typical withdrawal symptoms of smoking cessation were not reported (i.e. depression, anxiety, insomnia, irritability, hunger, constipation). Moreover, there were no reported serious adverse events (i.e. events requiring unscheduled visit to the family practitioner or hospitalisation) during the study.

**Table 4:** **Common adverse events reported by participants who completed all study visits**

Adverse Event (AE)	Study Visits			
	4-week	8-week	12-week	24-week
	no. pts reporting AEs/total no. pts (%)	no. pts reporting AEs/total no. pts (%)	no. pts reporting AEs/total no. pts (%)	no. pts reporting AEs/total no. pts (%)
Throat/mouth irritation*	16/45 (35.6%)	9/43 (20.9%)	7/41 (17.1%)	4/38 (10.5%)
Dry throat/mouth	13/45 (28.9%)	10/43 (23.3%)	9/41 (22.0%)	7/38 (18.4%)
Headache	12/45 (26.7%)	9/43 (20.9%)	8/41 (19.5%)	8/38 (21.1%)
Dry cough	10/45 (22.2%)	7/43 (16.3%)	5/41 (12.2%)	2/38 (5.3%)
Dizziness^§^	7/45 (15.6%)	7/43 (16.3%)	5/41 (12.2%)	3/38 (7.9%)
Nausea	6/45 (13.3%)	5/43 (11.6%)	5/41 (12.2%)	5/38 (13.2%)
Sore throat	4/45 (8.9%)	3/43 (7.0%)	1/41 (2.4%)	1/38 (2.6%)
Palpitations	3/45 (6.7%)	3/43 (7.0%)	0/41 (0%)	0/38 (0%)
Choking sensation	2/45 (4.4%)	1/43 (2.3%)	0/41 (0%)	0/38 (0%)

*Throat and mouth irritation were described either as tickling, itching, or burning sensation.

^§^Dizziness, was also used to mean vertigo and light-headedness.

#### Product preferences

The PV users rated scores well above the mean for satisfaction and for helpfulness (enabling them to refrain from smoking), their mean (±SD) VAS values being 6.7 (±2.6) and 7.4 (±2.9) respectively. Moreover, participants recommended the use of PVs to friends or relatives who wanted to stop/reduce smoking, the mean (±SD) VAS value being 8.1 (±2.3). Predictably, PVs rated even higher scores when these summary statistics were computed with the exclusion of the study failures. Conversely, products perception and acceptance by those who failed to remain abstinent or reduce smoking was poor; the mean (±SD) VAS values for satisfaction and for helpfulness being 2.0 (±1.2) and 0.8 (±1.1), respectively. As expected, these individuals were unlikely to recommend PV use to friends or relatives; the mean (±SD) VAS value being 3.6 (±2.6).

The overall participants’ perception and acceptance of the product was good also because of its ease of use and general lack of technical malfunctions. Only three study participants could not use the product as recommended and were retrained. One participant reported a faulty atomizer, and two had faulty chargers; replacements were given to them.

## Discussion

Efficacy and safety of second generation PVs in long-term smoking cessation and/or smoking reduction studies have never been investigated. Here, we show for the first time that use of a second generation PV substantially decreases cigarette consumption without causing significant side effects in smokers not intending to quit. Participants were enthusiastic about using these products, the majority (i.e. 76%) completing the study with an overall quit rate of 36%. A further 30% of the participants were able to sustain ≥50% cigarette by the end of the study.

These preliminary findings are of great significance in view of the fact that all smokers in the study were, by inclusion criteria, not interested in quitting. Moreover, though not directly comparable with standard smoking cessation and/or reduction studies because of its design, success rates in the present study are not only higher than those obtained with pharmaceutical products for the treatment of nicotine addiction [23, 24], but also greater than those of first generation “cig-alikes” [14, 22]. In particular, comparison of current results with those obtained in a similar prospective 6-month pilot study with first generation “cig-alikes” published a few years ago [22] shows improvement in quit rates with PVs (22.5% for “cig-alike” users vs 36% for PVs users).

An explanation for the large success rate may be attributed to the high level of satisfaction with the performance of second generation PVs as cigarette substitutes. The high-capacity lithium battery did not require frequent recharge, and the efficient vaporizing systems allowed an uninterrupted vaping experience with sufficient number of puffs through the whole day. Moreover, the reliability of the PVs under investigations was more than satisfactory with only a few reported technical malfunctions. Although not specifically measured in this study, nicotine absorption using second-generation devices has been shown to be consistently superior compared to “cig-alikes” [17, 18]. The high level of satisfaction with the product under investigation is substantiated by the notion that 30 out of the 38 who attended the last study visit were still using their PVs. This together with the high retention rate and elevated rating in likeability scores indicates that quality and attractiveness of the study product may be playing a vital role in attaining large success rates. Nonetheless, no correlations were observed between daily consumption of e-Liquid and success rates suggesting that multiple mechanisms are at play.

Throat/mouth irritation, and dry throat/mouth, headache and dry cough were frequently reported at the beginning of the study. These are likely to be secondary to exposure to PG mist generated by the PVs; exposure to PG mist may occur from smoke generators in discotheques, theatres, and aviation emergency training and is known to cause ocular, mouth, throat, upper airway irritation and cough [25, 26]. However, with the exception of headache (a highly unspecific symptom), the irritative symptoms – and particularly dry cough - appeared to wane spontaneously with time. Of note, typical withdrawal symptoms of smoking cessation were not reported during the course of the study. It is possible that the PVs under investigation by providing a coping mechanism for conditioned smoking cues could mitigate withdrawal symptoms associated with smoking reduction and smoking abstinence. Dizziness, nausea and palpitations may be due to nicotine overdosing, but these were not common and substantially declined with time. The substantial reduction in the frequency of dizziness and the lack of reported palpitation at later time points may be due to the improved familiarisation with the puffing technique and/or to individual adjustments/reductions in e-liquid consumption. In contrast from other ENDDs such some heat-and-burn platforms that can generate toxic levels of eCO [27], the products under investigation lead to substantial reduction in eCO levels, as expected in this vapour category [14, 22, 28]. Although larger and longer studies will be required for a full assessment of their adverse events, the present findings add to the current evidence that vaping is by far a less harmful alternative to tobacco smoking [29, 30].

There are some limitations in our study. Firstly, this was a small uncontrolled study, hence the results should be interpreted with caution. However, it would have been quite problematic to have a placebo arm in a study in which smokers were not interested in quitting. Secondly, 32.5% of the participants failed to attend their final follow-up visit, but this is not unexpected in a smoking cessation study and study outcome measures were computed by intention-to-treat analysis. Thirdly, because of its unusual design (smokers not willing to quit, PVs were used throughout the entire study period) this is not an ordinary cessation study and therefore direct comparison with other smoking cessation products cannot be made. Fourthly, assessment of withdrawal symptoms in our study was not rigorous and it is likely that was liable to recall bias. Therefore, the reported lack of withdrawal symptoms in the study participants should be considered with caution. Lastly, because only a single nicotine strength (i.e. 9 mg/mL) and a single aroma (i.e. tobacco flavour) were investigated in this study, it is possible that we failed to further maximize success rates. It is now known that unrestricted access to a wider selection in e-Liquid nicotine strength and flavour variability play a pivotal role in the attractiveness and success rates of these products [31, 32].

## Conclusions

Complete tobacco cessation is the best outcome for smokers, but the powerful addictive qualities of nicotine and of the ritualistic behavior of smoking create a huge hurdle, even for those with a strong desire to quit. Tobacco harm reduction (THR), the substitution of low-risk nicotine products for cigarette smoking, is a realistic strategy for smokers who have difficulty quitting. E-cigarettes are the newest and most promising products for THR [33]. This approach has been recently exploited to reduce or reverse the burden of harm in smokers with mental health disorders and chronic airway disease [34, 35].

E-cigarette is an attractive long-term alternative and safer source of nicotine to conventional cigarette [11, 12]. Since their invention in 2003, there has been constant innovation and development of more efficient and appealing products. Here we show for the first time that second generation PVs can substantially decrease cigarette consumption without causing significant side effects in smokers not intending to quit. Moreover, overall participants’ perception and acceptance of these products was very good, in particular for those who quit or reduced smoking. Compared to our earlier work with first generation “cig-alikes” [14, 22], technical problems and difficulties in use familiarization with second generation PVs were negligible. Improved products reliability and attractiveness might have contributed to the very low number of study failures and lost to follow-up and high success rates thus confirming the notion that these products are attractive substitutes for conventional cigarettes. Although large and carefully conducted RCTs will be required to confirm these preliminary encouraging observations, the notion that second generation PVs can substantially decrease cigarette consumption in smokers not intending to quit should be taken into consideration by regulatory authorities seeking to adopt proportional measures for the vapour category [36].

## Abbreviations

e-Cig: Electronic-Cigarette
ENDD: Electronic nicotine delivery device
Cig/day: Cigarettes smoked per day
mmHg: Millimetres of mercury
FTND: Fagerstrom test of nicotine dependence
BDI: Beck’s depression inventory
eCO: Exhaled carbon monoxide
mg: Milligrams
VAS: Visual analogue score
ppm: Parts per million
Pack/yrs: Pack-years
SD: Standard deviation
IQR: Interquartile range.
